# Propylthiouracil-related Toxic Hepatitis: Impact of Silent Cases

**DOI:** 10.5005/jp-journals-10018-1153

**Published:** 2016-07-09

**Authors:** Murat Eser, Sebahat Basyigit, Mithat Eser, Yasar Nazligul

**Affiliations:** 1 Department of Internal Medicine, Kecioren Research and Training Hospital, Ankara, Turkey; 2 Department of Gastroenterology, Kecioren Research and Training Hospital, Ankara, Turkey; 3 Department of Internal Medicine, Gulhane Millitary Medical Academy, Etlik, Turkey

**Keywords:** Liver function tests, Propylthioutacil, Toxic hepatitis.

## Abstract

Liver is the main organ which can metabolize many drugs or chemical agents. Toxic events developed by drugs are one of the most common causes of liver damage. Toxic hepatitis can be encountered in different clinical situations, such as acute hepatitis, fulminant hepatitis chronic hepatitis or cirrhosis. We aimed to report a case of asymptomatic toxic hepatitis in a patient taking propylthiouracil (PTU). A 38 years old female patient admitted to hospital complained of fatigue. She had no special medical history except Graves’ disease. She had been taking PTU 300 mg/day for 1 month. She had no history of another medication, eating mushroom, alcohol consumption, traveling, family history of liver disease. Her physical examination was normal. Laboratory analysis revealed that alanine aminotransferase—543 U/L, aspartate aminotransferase—227 U/L, gamma glutamyl transferase—66 U/L and alkaline phosphatase—136 U/L. Serum levels of bilirubin and albumin, INR, complete blood count and thyroid function tests were all normal. She had normal liver function test (LFT) before using PTU. Propylthiouracil was discontinued and she was given methimazole. She was examined for the etiology of abnormal LFT, but no specific etiology could be recorded. She was thought to have toxic hepatitis related to PTU. In her follow-up LFT has turned to normal level (Table 1).

**How to cite this article:**

Eser M, Basyigit S, Eser M, Nazligul Y. Propylthiouracil-related Toxic Hepatitis: Impact of Silent Cases. Euroasian J Hepato-Gastroenterol 2015;5(2):134-135.

Dear Editor,

Liver is the main organ which can metabolize many drugs or chemical agents. Toxic events developed by drugs are one of the most common causes of liver damage. Toxic hepatitis can be encountered in different clinical situations, such as acute hepatitis, fulminant hepatitis chronic hepatitis or cirrhosis. We aimed to report a case of asymptomatic toxic hepatitis in a patient taking propylthiouracil (PTU). A 38 years old female patient admitted to hospital complained of fatigue. She had no special medical history except Graves’ disease. She had been taking PTU 300 mg/day for 1 month. She had no history of another medication, eating mushroom, alcohol consumption, traveling, family history of liver disease. Her physical examination was normal. Laboratory analysis revealed that alanine aminotransferase—543 U/L, aspartate aminotransferase—227 U/L, gamma glutamyl transferase—66 U/L and alkaline phosphatase—136 U/L. Serum levels of bilirubin and albumin, INR, complete blood count and thyroid function tests were all normal. She had normal liver function test (LFT) before using PTU. Propylthiouracil was discontinued and she was given methimazole. She was examined for the etiology of abnormal LFT, but no specific etiology could be recorded. She was thought to have toxic hepatitis related to PTU. In her follow-up LFT has turned to normal level (Table 1).

Propylthiouracil is an antithyroid agent that has been used for treatment of hyperthyroidism since 1940. Hepatotoxicity is the well known side effect of PTU. Severe liver damage is rare but life-threatening. The estimated incidence of PTU-related acute liver damage is 10%, fulminant hepatic failure is 1% in US. Mortality rate of acute liver failure related with PTU has been reported as high as 25%.^1^

**Table 1:** **Table 1:** Laboratory data of patients during the follow-up

		*ALT(U/L)*		*AST(U/L)*	
At the time of admission		575		253	
Day 1		465		190	
Day 5		233		77	
Day 15		136		51	
Day 25		35		47	

Abbrevations: ALT: Alanine aminotransferase; AST: Aspartate aminotransferase.

Although idiosyncrasy or immunological mechanisms are hold responsible for pathogenesis, the exact mechanism is unrecognized. Propylthiouracil-induced acute liver failure may develop at any age. Two-third of them are women because of Graves’ disease is dominant in women.^2^

There is no defined risk factor for developing hepa-totoxicity. It is independent from duration of treatment. The liver function tests are normalized in most patients between 16 and 145 days after discontinuation of PTU. But liver damage can also be irreversible in some cases.^2^ There is also no estimated prognostic factor for assessing hepa-totoxicity. The simple way to prevent dramatic clinical conditions is monitoring the LFT and discontinuing the treatment when the hepatotoxicity is detected. Although the current guideline for treatment of hyperthyroidism suggests that patients should have a baseline LFT prior to anti-thyroid treatment, it does not recommend periodic LFT monitarization during treatment.^3^ Propylthiouracil induced liver failure presenting with obvious clinical signs has been highlighted in the literature repeatedly. The present case represents a PTU-related asymptomatic acute liver damage and support the significance of LFT moni-torization to detect liver damage and to reduce fatality.
